# Supplementary material for: Identification and Genetic Characterization of MERS-Related Coronavirus Isolated from Nathusius’ Pipistrelle (Pipistrellus nathusii) near Zvenigorod (Moscow Region, Russia)
Source: Int J Environ Res Public Health. 2023 Feb 19;20(4):3702. doi: 10.3390/ijerph20043702 (PMC9965006; doi:10.3390/ijerph20043702)
Supplement: Supplementary file 1 [file ijerph-20-03702-s001.zip › ijerph-2119089-supplementary.pdf]

International Journal of
Environmental Research and Public Health

MDPI

Table S1. Analysis of full genome identify among MOW-BatCoV 15-22 and other members of Merbecovirus (the top 10 of closest full genome sequences available in GenBank).

Description	Given Scientific Name (by Author's)	Host	Collection date	Country	Query Cover	Per. ident	Acc. Len	Accession
Bat-CoV/H.savii/Italy/206645-40/2011, complete genome	Middle East respiratory syndrome-related coronavirus	*Hypsugo savii* (bat)	2011	Italy	88%	81,48%	30048	MG596802.1
Bat-CoV/P.khulii/Italy/206645-63/2011, complete genome	Middle East respiratory syndrome-related coronavirus	*Pipistrellus kuhlii* (bat)	2011	Italy	88%	81,45%	30039	MG596803.1
Bat coronavirus Vs-CoV-1 genomic RNA, nearly complete genome	Bat coronavirus	*Vespertilio sinensis* (bat)	un-described	Japan: Tokyo, Okutama	85%	81,32%	29930	LC469308.1
BtVs-BetaCoV/SC2013, complete genome	BtVs-BetaCoV/SC2013	*Vespertilio superans* (bat)	2013	China	82%	81,27%	30423	KJ473821.1
isolate PREDICT/PDF-2180, complete genome	Bat coronavirus	*Pipistrellus cf. hesperidus* (bat)	2013	Uganda	82%	80,84%	29642	NC_034440.1
isolate Riyadh_9_2013, complete genome	Middle East respiratory syndrome-related coronavirus	*Homo sapiens*	2013	Saudi Arabia	82%	82,47%	30055	KJ156869.1
isolate Hu/Jordan-201440011123/2014, complete genome	Middle East respiratory syndrome-related coronavirus	*Homo sapiens*	2014	Jordan	82%	82,46%	30123	MK039552.1
strain Hu/UAE_032_2014, complete genome	Middle East respiratory syndrome-related coronavirus	*Homo sapiens*	2014	United Arab Emirate	82%	82,46%	30123	KY581693.1
camel/UAE_B73_2015, complete genome	Middle East respiratory syndrome-related coronavirus	*Camelus dromedarius*	2015	United Arab Emirates	82%	82,46%	30123	MF598663.1
isolate Florida/USA-2_Saudi Arabia_2014, complete genome	Middle East respiratory syndrome-related coronavirus	*Homo sapiens*	2014	USA	82%	82,46%	30123	KP223131.1
